# Sugar-sweetened beverage consumption among Indigenous Australian children aged 0–3 years and association with sociodemographic, life circumstances and health factors

**DOI:** 10.1017/S1368980019001812

**Published:** 2019-08-28

**Authors:** Katherine A Thurber, Johanna Long, Minette Salmon, Adolfo G Cuevas, Raymond Lovett

**Affiliations:** 1 National Centre for Epidemiology and Population Health, Research School of Population Health, Australian National University, 54 Mills Road, Acton, ACT 2602, Australia; 2 Medical School, Australian National University, Acton, ACT, Australia; 3 Department of Community Health, Tufts University, Medford, MA, USA

**Keywords:** Sugar-sweetened beverages, Feeding behaviour, Indigenous health, Child health, Water quality

## Abstract

**Objective:**

To explore beverage intake and associations between sugar-sweetened beverage (SSB) intake and sociodemographic, life circumstances, health and well-being factors in a national cohort of Indigenous children.

**Design:**

We calculated prevalence ratios for any SSB consumption across exposures, using multilevel Poisson regression (robust variance), adjusted for age group and remoteness. A key informant focus group contextualised these exploratory findings.

**Setting:**

Diverse settings across Australia.

**Participants:**

Families of Indigenous children aged 0–3 years, in the Longitudinal Study of Indigenous Children.

**Results:**

Half (50·7 %, *n* 473/933) of children had ever consumed SSB at survey, increasing from 29·3 % of 0–12-month-olds to 65·7 % of 18–36-month-olds. SSB consumption prevalence was significantly lower in urban and regional *v*. remote areas, and in families experiencing socio-economic advantage (area-level advantage, caregiver employed, financial security), better life circumstances (caregiver social support, limited exposure to stressors) and caregiver well-being (non-smoking, social and emotional well-being, physical health). SSB consumption prevalence was significantly lower among those engaged with health services (adequate health-service access, regular prenatal check-ups), except SSB consumption prevalence was higher among those who received home visits from an Aboriginal Health Worker compared with no home visits. Key informants highlighted the role of water quality/safety on SSB consumption.

**Conclusions:**

A substantial proportion of Indigenous children in this sample consumed SSB from an early age. Health provider information needs to be relevant to the context of families’ lives. Health system strategies must be paired with upstream strategies, such as holistic support programmes for families, reducing racism and improving water quality.

Nutrition in the first years of life is critical to early childhood health and development^(^^1^^–^^3^^)^. Australian Infant Feeding Guidelines recommend breast milk as the sole source of nutrition for children’s first 6 months, where possible; breast-feeding of any duration benefits both the child and mother^(^^4^^)^. From age 6 months to 3 years, guidelines recommend slow introduction of water and other micronutrient-dense foods and drinks, alongside continued breast-feeding as long as the child and mother desire. Guidelines recommend avoiding foods and drinks containing added sugar^(^^4^^)^.

Caregivers^(^^5^^)^ and health practitioners, including Aboriginal Health Workers (AHW)^(^^5^^–^^7^^)^, have expressed concerns about sugar-sweetened beverage (SSB) consumption by young Aboriginal and Torres Strait Islander children. SSB are high in sugar and contain limited, if any, nutritional value. A large body of research has demonstrated detrimental health impacts of SSB consumption including dental caries, obesity, hypertension, type 2 diabetes and CVD in children and adults^(^^8^^–^^14^^)^. Early exposure to SSB can also contribute to enduring preferences for sweet-tasting foods and beverages^(^^15^^–^^17^^)^.

There is a high prevalence of SSB consumption in Australia, and particularly high consumption prevalence and volume among Aboriginal and Torres Strait Islander peoples^(^^18^^–^^20^^)^. While the measurement approaches and populations have varied across studies, there is consistent localised and national quantitative evidence of high SSB intake by Aboriginal and Torres Strait Islander children in the first 3 years of life (Table 1)^(^^18^^,^^21^^–^^26^^)^. For example, data from the National Aboriginal and Torres Strait Islander Nutrition and Physical Activity Survey (NATSINPAS) indicated that 26·0 (95 % CI 13·5, 38·5) % of Aboriginal and Torres Strait Islander children aged 2–3 years had consumed cordial on the day prior to interview, compared with 10·3 (95 % CI 6·4, 14·2) % of non-Indigenous children in the Australian Health Survey^(^^18^^,^^21^^,^^22^^)^. The gap in consumption prevalence is narrower at older ages; data are not available for children less than 2 years of age.

**Table 1:** Synthesis of previous evidence on sugar-sweetened beverage (SSB) and other beverage consumption by Aboriginal and Torres Strait Islander children aged 0–3 years

Source	Sample characteristics					% consuming each beverage type										
	Year(s) of data collection	Geographic area	Remoteness	Age (months)	Sample size	Any SSB	Cordial	Soft drinks	Sweetened tea/coffee	Fruit juice	Water	Still breast-fed	Formula	Cow’s milk	Flavoured milk	Other
Eades *et al.*^(^^24^^)^*	Mid-1990s	Perth, WA	Urban	7–8	166	–	37	12	5	53	61	33	48	16	15	13
				12	169	–	60	33	17	70	65	23	18	50	33	23
Leonard *et al.*^(^^23^^)^†	2010–2012	Northern Australia	Remote	6–11	122	–	–	12	–	–	–	80	22	–	–	–
				12–17	62	–	–	37	–	–	–	53	3	–	–	–
				18–23	43	–	–	44	–	–	–	51	5	–	–	–
Ashman *et al.*^(^^25^^)^‡	2010	NSW	1 rural town and 1 remote town	3	25	–	–	–	–	8	16	24	88	4	–	0
				6	17	–	–	–	–	18	59	29	71	6	–	6
				9	14	–	–	–	–	21	79	36	71	14	–	7
				12	23	–	–	–	–	48	83	9	48	48	–	26
Australian Bureau of Statistics^(^^21^^,^^22^^)^§	2012–13	National (representative)	Urban, regional and remote	24–36	∼300	53	26	18	3	19	68	–	4	89	4	12
Cockburn *et al.*^(^^19^^)^‖	2010–2012	National (not representative)	Urban, regional	0–23	230	–	4		–	64	–	–	–	–	–	9
				24–47	179/180	–	49		–	69	–	–	–	–	–	10

* Beverage infants were ‘mostly or ‘sometimes’ given in their bottles. ‘Cool drink’ classified under ‘Soft drinks’. Other drink refers to ‘powdered milk’.

† Infant intake the previous day. ‘Sweet drinks’ classified under ‘Soft drinks’.

‡ Infant intake in past 24 h. Other drink refers to ‘Sweetened/flavoured water’.

§ Child intake in past 24 h. The survey included 356 children aged 2–4 years (data on exact number aged 2–3 years unavailable), for a weighted estimate of 47 600 children. ‘Tea’ and ‘Coffee, coffee substitutes’ are included under ‘Sweetened tea/coffee’, but the survey did not ask whether or not these drinks were sweetened. ‘Fruit and vegetable drinks’ are classified under ‘Fruit juice’. ‘Soft drinks and flavoured mineral waters’ are classified under ‘Soft drinks’. ‘Dairy milk (cow, sheep and goat)’ are classified under ‘Cow’s milk’. ‘Flavoured milks and milk shakes’ are classified under ‘Flavoured milk’. ‘Other’ drink refers to ‘Other beverage flavourings and prepared beverages’.

‖ Infant intake in the past 24 h. Data on ‘Soft drink and cordial’ are combined. ‘Other’ drink refers to ‘Diet soft drink and cordial’.

Before colonisation, exclusive breast-feeding for at least 6 months, and extended breast-feeding with gradual introduction of micronutrient-dense foods, commonly occurred^(^^6^^,^^24^^,^^27^^–^^29^^)^. Colonisation disconnected Aboriginal and Torres Strait Islander people from their family, land, culture and traditional knowledge, and has contributed to ongoing trauma^(^^30^^,^^31^^)^. Colonial practices such as relocating Aboriginal and Torres Strait Islander people to missions, the provision of food rations and separating mothers from their children interrupted intergenerational knowledge transfer, contributing to the loss of knowledge about infant feeding practices^(^^5^^–^^7^^,^^29^^,^^32^^,^^33^^)^. Further, the colonisation of Australia led to persisting social, economic, health and structural inequities for Aboriginal and Torres Strait Islander peoples^(^^30^^,^^34^^)^. As a result, many Aboriginal and Torres Strait Islander families experience life circumstances, stressors or health conditions that may preclude adherence to child dietary recommendations^(^^5^^,^^31^^)^.

There is substantial potential for health gain through reducing SSB intake by young Aboriginal and Torres Strait Islander children, particularly given the disproportionate burden of obesity and dental caries experienced by this population^(^^35^^)^. To inform policy that supports healthy feeding practices in Aboriginal and Torres Strait Islander families, it is important to understand the factors underlying SSB consumption in the first years of life^(^^18^^)^. Factors including low maternal education, financial insecurity, overcrowding, poor maternal physical or social and emotional well-being, exposure to stressors, culturally inappropriate advice and programmes, and inadequate social support may pose barriers to optimum infant feeding^(^^5^^,^^6^^,^^25^^,^^31^^,^^32^^,^^36^^,^^37^^)^. However, we lack quantitative evidence of the association between social, cultural and environmental factors and SSB intake in this population, nationally and across levels of remoteness^(^^18^^)^. The current exploratory study aimed to: (i) quantify the prevalence of any SSB intake and the relationships between SSB intake and a range of sociodemographic, life circumstances, and health and well-being factors in a national cohort of Aboriginal and Torres Strait Islander children aged 0–3 years; and (ii) contextualise these quantitative findings by engaging with a group of Aboriginal and Torres Strait Islander key informants, to support a participatory approach to analysis.

## Methods

### Study design and participants

The present study analysed quantitative data from the Longitudinal Study of Indigenous Children (LSIC), an ongoing national study funded and managed by the Australian Government Department of Social Services. LSIC was designed through extensive consultation with Aboriginal and Torres Strait Islander peoples^(^^38^^)^. Aboriginal and Torres Strait Islander children were recruited from eleven diverse sites across Australia using purposive sampling. These sites were chosen to reflect the diversity of environments in which Aboriginal and Torres Strait Islander children live^(^^39^^)^. Two age cohorts of children were recruited to participate in the study: the B cohort (intended age 0·5–1·5 years) and the K cohort (intended age 3·5–4·5 years); the present analysis is based on data from families of children in the B cohort who participated in Wave 1 of the study in 2008 (*n* 954). Further details of LSIC’s design are provided elsewhere^(^^38^^,^^39^^)^.

Research Administration Officers (RAOs) conduct annual face-to-face interviews with children and their primary caregiver (hereafter referred to as ‘caregiver’). In most cases, the caregiver is the study child’s mother, but can also be the child’s father, stepmother, stepfather, relative or another guardian. The caregiver reported all data included in the current analysis except remoteness and area-level advantage, which were derived from participants’ addresses. In cases where the caregiver was not the child’s birth mother, questions about the pregnancy and study child’s birth were asked about the child’s birth mother.

### Sample

Children were excluded from analysis if they were over 3 years of age (*n* 8/954) or they were missing data on the outcome and/or on remoteness (*n* 13/946); the final sample included 933 families.

### Variables

#### Exposures

The online supplementary material, Supplemental File S1, provides a detailed description of exposure variables (sociodemographic, life circumstances, and health and well-being factors).

#### Outcome: sugar-sweetened beverage consumption

Caregivers were asked about the child’s breast-feeding history, the child’s age at first consumption of other forms of milk or formula, and the first type of non-breast milk or formula consumed. Caregivers were then asked, ‘What does (study child) drink now?’ Interviewers recorded all responses provided by the caregiver and categorised these as: hasn’t had anything else yet, formula, cow’s milk (carton/bottle), long-life milk, powdered milk (dried, Sunshine), tinned milk (Carnation), coconut milk, flavoured milk, water, fruit juice (hereafter referred to as ‘fruit drink’), cordial, fizzy drink (hereafter referred to as ‘soft drink’), sweetened tea/coffee and other. Interviewers only read out the option(s) if necessary to clarify a caregiver’s response.

We classified cordial, soft drink and/or sweetened tea/coffee as SSB. Fruit drink was not included in this definition; as the sugar content of these beverages was not recorded in the survey, it was not possible to differentiate between 100 % fruit juice and fruit drink with added sugars.

While a time period was not specified in this question, based on the sequence of interview questions, responses were interpreted to represent the types of drinks children had been consuming since the introduction of any non-breast-milk drinks, therefore providing a measure of caregiver-reported infant ‘ever’ SSB consumption.

### Analytical methods

We conducted descriptive analysis of SSB and other beverage consumption, overall and by age group and remoteness. We conducted *χ*^2^ tests to test for an association between beverage consumption prevalence and age group and level of remoteness.

We calculated prevalence ratios (PR) using multilevel Poisson models with robust variance to quantify the association between exposure variables and SSB, accounting for within-cluster correlation, and adjusted for age group and remoteness. The Wald statistic was also used to test the association between SSB and exposure variables as a whole. For ordinal variables (child age, area-level advantage, number of homes lived in since birth, household size, number of negative life events), we tested for trend by re-running models with the exposure coded as a continuous variable.

Given the potential for residual confounding by remoteness, we tested for an interaction by and repeated analyses stratified by remoteness (urban/inner regional *v*. outer regional/remote, including remote and very remote; online supplementary material, Supplemental File S2).

### Participatory research methods

Aligned with the participatory research methodology underpinning LSIC, the present analysis of quantitative data from LSIC incorporates participatory research approaches^(^^40^^)^. In July 2018, the lead author (non-Indigenous) conducted a knowledge exchange discussion (focus group) with the RAOs (*n* 7) currently conducting LSIC interviews. All RAOs are Aboriginal and/or Torres Strait Islander, and most live in the area in which they conduct interviews. Not all of the current RAOs were involved in Wave 1 data collection, but we consider the RAOs to be key informants, holding valuable contextual knowledge about these communities and participating families^(^^41^^)^. Many of the RAOs have been engaged with these families and communities over multiple years and have developed strong and trusting relationships^(^^40^^)^. The primary aims of the focus group were to share preliminary findings, discuss potential interpretations and key messages, inform further analyses, and assist with developing a feedback sheet for LSIC participants. The focus group was semi-structured; K.A.T. presented early findings, and asked participants to reflect on each finding and provide their interpretations. Participants were encouraged to share their experiences and perspectives and discuss differences across contexts. Learnings from the focus group are incorporated into the ‘Discussion’ section.

## Results

The sample included 473 males (50·7 %) and 460 females (49·3 %), with 30·3 % aged 0–12 months (*n* 283), 41·6 % aged 12–18 months (*n* 388) and 28·1 % aged 18–36 months (*n* 262). Close to a third of participants each lived in major cities (27·4 %, *n* 256), regional areas (37·1 %, *n* 346) and remote areas (35·5 %, *n* 331).

### Beverage consumption

Half (50·7 %, *n* 473/933) of children had consumed any SSB; 46·8 % (*n* 437) had consumed cordial, 19·2 % (*n* 179) soft drink and 12·7 % (*n* 118) sweetened tea/coffee (Table 2). The prevalence of consumption of any SSB and individual SSB types was higher in older compared with younger age groups, and in more remote compared with regional and urban areas, although it was high in all settings.

**Table 2:** Sugar-sweetened beverage (SSB) and other beverage consumption by Aboriginal and Torres Strait Islander children aged 0–3 years in the Longitudinal Study of Indigenous Children Wave 1 (2008), overall and by age group and level of remoteness

			By age group						By level of remoteness					
	Overall (*N* 933)		0–12 months (*N* 283)		>12–18 months (*N* 388)		>18–36 months (*N* 262)		Major cities (*N* 256)		Regional (*N* 346)		Remote (*N* 331)	
	%	*n*	%	*n*	%	*n*	%	*n*	%	*n*	%	*n*	%	*n*
Any SSB†,‡	50·7	473	29·3	83	56·2	218	65·7	172	36·3	93	49·7	172	62·8	208
Cordial†,‡	46·8	437	26·9	76	52·1	202	60·7	159	32·8	84	46·5	161	58·0	192
Fizzy drinks†,‡	19·2	179	5·7	164	20·9	81	31·3	82	10·2	26	19·1	66	26·3	87
Sweetened tea/coffee†,‡	12·7	118	7·1	20	12·4	48	19·1	50	5·5	14	6·4	22	24·8	82
Fruit drink†,‡	75·6	705	65·0	18	78·9	306	82·1	215	68·8	176	73·1	253	83·4	276
Water†,‡	85·4	797	80·6	228	87·9	341	87·0	228	87·9	225	78·9	273	90·3	299
Still breast-fed*,†,‡	26·3	244	33·6	95	26·6	102	18·1	47	14·1	36	15·6	54	47·0	154
Formula†,‡	30·0	280	49·8	141	26·0	101	14·5	38	41·4	106	27·5	95	23·9	79
Cow’s milk†,‡	56·0	522	30·0	85	63·1	245	73·3	192	53·1	136	62·4	216	51·4	170
Flavoured milk†,‡	24·5	229	12·0	34	28·1	109	32·8	86	17·6	45	28·6	99	25·7	85
Other milk†,‡	11·4	106	6·4	18	12·9	50	14·5	38	2·3	6	5·8	20	24·2	80

Other milk includes long-life, powdered, tinned and coconut milk.

* *N* 6 missing data on breast-feeding duration; total *N* is 283, 384 and 260 across age groups, and 253, 346 and 328 across levels of remoteness.

† Significant association with age group (*χ*^2^ test, *P* < 0·05).

‡ Significant association with remoteness (*χ*^2^ test, *P* < 0·05).

Most children had consumed water (85·4 %, *n* 797) and fruit drink (75·6 %, *n* 705); 26·3 % (*n* 244) were still being breast-fed, 30·0 % (*n* 280) had consumed formula, 56·0 % (*n* 522) cow’s milk, 24·5 % (*n* 229) flavoured milk and 11·4 % (*n* 106) other types of milk. Consumption prevalence of fruit drink, water, cow’s milk, flavoured milk and other milk was higher in older age groups; prevalence of breast-feeding and formula consumption was lower in older age groups. Consumption of fruit drink (70–80 %), water (80–90 %), cow’s milk (50–60 %) and flavoured milk (20–30 %) was common across levels of remoteness. Consumption of formula was more common in major cities (41·4 %, *n* 106/256) compared with regional (27·5 %, *n* 95/346) or remote (23·9 %, *n* 79/331) areas, and the prevalence of breast-feeding and consumption of other forms of milk (long-life milk, powdered, tinned and coconut) was substantially higher among children in remote areas (47·0 %, *n* 154/328 and 24·2 %, *n* 80/331, respectively) compared with major cities (14·2 %, *n* 36/253 and 2·3 %, *n* 6/256) and regional areas (15·6 %, *n* 54/346 and 5·8 %, *n* 20/346).

### Association between exposures and children’s sugar-sweetened beverage consumption

#### Sociodemographic factors

SSB consumption increased with age and number of children in the household and decreased with increasing area-level advantage (*P*_trend_ < 0·05; Table 3). SSB consumption prevalence was significantly lower among children: in major cities and regional areas compared with remote areas who were Torres Strait Islander compared with Aboriginal; whose mother was 21–30 years or >30 years of age compared with ≤21 years at their birth; and whose caregiver was employed compared with not employed. We did not observe an association of SSB consumption with child gender or caregiver relationship status.

**Table 3:** Association of sugar-sweetened beverage (SSB) consumption by Aboriginal and Torres Strait Islander children aged 0–3 years with sociodemographic, life circumstances, and health and well-being factors (combined sample) in the Longitudinal Study of Indigenous Children Wave 1 (2008)

	% any SSB	*n*/*N*	PR	95 % CI	*P* value
Total	50·7	473/933	–	–	–
Sociodemographic factors					
Child gender					0·570
Male	49·9	236/473	1·00	Ref.	
Female	51·5	237/460	1·03	0·93, 1·13	
Child age group*					<0·001
0–12 months	29·3	83/283	1·00	Ref.	
>12–18 months	56·2	218/388	1·88	1·51, 2·33	
>18–36 months	65·7	172/262	2·13	1·66, 2·74	
Child Indigenous status					0·079
Aboriginal	51·7	419/811	1·00	Ref.	
Torres Strait Islander	35·0	21/60	0·63	0·42, 0·95	
Aboriginal & Torres Strait Islander	53·2	33/62	0·91	0·72, 1·14	
Area-level advantage*					0·377
Most advantaged	38·3	75/196	0·80	0·58, 1·10	
Mid-level advantage	51·1	298/583	0·88	0·67, 1·16	
Most disadvantaged	64·9	100/154	1·00	Ref.	
Remoteness					0·001
Major cities	36·3	93/256	0·63	0·49, 0·80	
Regional	49·7	172/346	0·85	0·68, 1·07	
Remote	62·8	208/331	1·00	Ref.	
Mother’s age at birth					0·002
Not birth mother	50·9	28/55	0·82	0·66, 1·03	
Up to 21 years	62·6	129/206	1·00	Ref.	
21–30 years	50·2	213/424	0·84	0·74, 0·96	
>30 years	41·5	103/248	0·73	0·62, 0·86	
Caregiver is partnered					0·938
No	50·9	208/409	1·00	Ref.	
Yes	50·6	265/524	1·00	0·89, 1·11	
Caregiver is employed					0·025
No	53·7	364/678	1·00	Ref.	
Yes	43·2	107/248	0·83	0·71, 0·98	
Missing	28·6	2/7	–	–	
Number of children in the household*					0·055
1	42·9	109/254	0·80	0·67, 0·97	
2	47·9	112/234	0·84	0·71, 1·00	
3	53·5	106/198	0·94	0·81, 1·09	
4 or more	59·1	146/247	1·00	Ref.	
Life circumstances					
Caregiver has a strong family					0·028
Not really or sometimes	58·2	78/134	1·00	Ref.	
Most times	43·4	56/129	0·76	0·62, 0·94	
Always	50·6	338/668	0·85	0·72, 0·99	
Missing	50·0	1/2	–	–	
Caregiver has lots of friends					0·005
None or not many	53·5	92/172	1·00	Ref.	
Fair few	53·3	170/319	1·03	0·88, 1·21	
Lots	47·7	211/442	0·81	0·68, 0·97	
Caregiver has someone to talk to when upset					0·059
Never or little bit	59·3	83/140	1·00	Ref.	
Fair bit	49·2	60/122	0·87	0·71, 1·07	
Always	49·1	328/668	0·83	0·71, 0·97	
Missing	66·7	2/3	–	–	
Caregiver advice from mothers, Aunties, Elders					0·559
No	49·7	298/600	1·00	Ref.	
Yes	53·2	148/278	1·04	0·91, 1·19	
Missing	49·1	27/55	–	–	
Family financial strain					0·001
We run out of money	64·3	101/157	1·00	Ref.	
We have just enough money	51·7	216/418	0·81	0·69, 0·95	
We can save	44·9	144/321	0·69	0·57, 0·84	
Missing	32·4	12/37	–	–	
Family worries about money					0·008
No	48·5	315/650	0·83	0·73, 0·95	
Yes	55·7	151/271	1·00	Ref.	
Missing	58·3	7/12	–	–	
Home needs major repairs					0·258
No	47·0	259/551	0·92	0·80, 1·06	
Yes	55·6	203/365	1·00	Ref.	
Missing	64·7	11/17	–	–	
Number of homes since study child’s birth					0·299
1	47·5	290/611	0·87	0·73, 1·05	
2	55·6	125/225	0·94	0·77, 1·14	
3 or more	60·2	53/88	1·00	Ref.	
Missing	55·6	5/9	–	–	
Caregiver experience of discrimination					0·016
Not really	51·8	281/543	0·87	0·77, 0·97	
Little bit to lots of times	59·1	133/225	1·00	Ref.	
Missing	35·8	59/165	–	–	
Humbugged in the last year					0·001
No	47·3	343/725	0·79	0·70, 0·91	
Yes	62·1	126/203	1·00	Ref.	
Missing	80·0	4/5	–	–	
Number of negative life events*					<0·001
0	39·9	59/148	0·68	0·55, 0·85	
1	44·5	101/227	0·73	0·64, 0·84	
2	47·7	82/172	0·80	0·67, 0·96	
3 to 10	61·4	202/329	1·00	Ref.	
Missing	50·9	29/57	–	–	
Health and well-being					
Prenatal check-ups					0·060
No check-ups	66·7	16/24	1·00	Ref.	
Some check-ups, not regular	63·6	28/44	0·93	0·69, 1·24	
Regular check-ups	49·6	400/807	0·80	0·63, 1·00	
Missing	50·0	29/58	–	–	
Smoking during pregnancy					<0·001
No	42·9	176/410	0·77	0·67, 0·89	
Yes	58·3	256/439	1·00	Ref.	
Missing	48·8	41/84	–	–	
Duration of breast-feeding					0·638
Never breast-fed	46·4	84/181	1·00	Ref.	
Less than 6 months	49·2	97/197	1·08	0·88, 1·33	
6 months or more	52·2	286/548	1·01	0·84, 1·21	
Missing	85·7	6/7	–	–	
Postnatal depression					0·251
No	50·6	367/726	0·94	0·76, 1·16	
Yes (treated)	55·4	36/65	1·09	0·83, 1·44	
Yes (untreated)	48·6	35/72	1·00	Ref.	
Missing	50·0	35/70	–	–	
Caregiver social and emotional well-being					<0·001
Good (low distress)	44·9	286/637	0·73	0·62, 0·85	
Poor (high distress)	65·3	113/173	1·00	Ref.	
Missing	60·2	74/123	–	–	
Child general health					0·799
Good, fair or poor	50·8	98/193	1·00	Ref.	
Excellent or very good	50·8	375/738	0·98	0·84, 1·14	
Missing	0·0	0/2	–	–	
Caregiver general health					0·014
Good, fair or poor	54·2	254/469	1·00	Ref.	
Excellent or very good	47·6	215/452	0·85	0·75, 0·97	
Missing	33·3	4/12	–	–	
Access to health services					0·003
Adequate access	48·9	415/848	0·76	0·64, 0·91	
Unmet need for health services	67·9	55/81	1·00	Ref.	
Missing	75·0	3/4	–	–	
Home visits after birth					<0·001
No home visits	52·7	187/355	1·00	Ref.	
Home visits not from AHW	45·7	210/460	1·00	0·85, 1·17	
Home visits from AHW	70·1	75/107	1·37	1·17, 1·59	
Missing	9·1	1/11	–	–	
Caregiver currently smokes					<0·001
No	40·6	159/392	0·76	0·63, 0·85	
Yes	58·0	314/541	1·00	Ref.	
Caregiver knowledge of family, culture, history					0·886
Not much or a little bit	49·3	150/304	1·00	Ref.	
Fair bit	49·8	124/249	0·97	0·83, 1·14	
Lots	52·7	199/378	0·96	0·81, 1·13	
Missing	0·0	0/2	–	–	

PR, prevalence ratio; AHW, Aboriginal Health Worker; ref., reference category.

All models exclude children missing data on the exposure of interest; all models are adjusted for age group and remoteness (where appropriate).

*P* value shown is for the Wald statistic.

The missing category for caregiver discrimination is large because caregivers were asked this question only if they identified as Aboriginal and/or Torres Strait Islander.

* Significant trend of decreasing prevalence of SSB intake with decreasing value of the exposure variable, i.e. decreasing prevalence with decreasing area-level disadvantage, household size and number of negative life events (*P*_trend_ < 0·05); only tested for ordinal variables.

#### Life circumstances factors

SSB consumption prevalence was significantly lower for children whose caregivers had higher *v*. lower levels of instrumental support (caregiver has a strong family), social network (caregiver has lots of friends) and emotional support (caregiver has someone to talk to when upset; Table 3). SSB consumption prevalence was significantly lower among children whose families were financially secure (lower financial strain, no worries about money) and who were exposed to fewer stressors (limited exposure to racism, not humbugged,* low number of negative major life events in the past year). We did not observe an association between SSB and maternal informational support (caregiver receipt of pregnancy and birthing advice from mothers, Aunties or Elders) or housing (home needs repairs; number of homes lived in since birth).

#### Health and well-being factors

SSB consumption prevalence was significantly lower among children who were not exposed to smoke *in utero*, whose caregiver had good social and emotional well-being and physical health, whose mother had regular compared with no prenatal check-ups, who had adequate access to health services and whose caregiver was not a current smoker (Table 3).

SSB consumption prevalence was significantly higher among children whose family received postnatal home visits from an AHW compared with no home visits. We did not observe an association of SSB consumption prevalence with breast-feeding duration, postnatal depression, child physical health or caregiver cultural knowledge.

### Differences between urban/inner regional and remote/outer regional settings

We observed a difference between urban/inner regional and remote/outer regional settings (*P*_interaction_ < 0·05) in the association between SSB and age group, caregiver relationship status, caregiver employment status, informational support, financial strain, worries about money, number of homes, negative life events, smoking during pregnancy, breast-feeding duration, caregiver current smoking and unmet need for health services (online supplementary material, Supplemental Table S1). In many cases, the pattern of association was similar, but the magnitude of effect was greater within the urban/inner regional sample and non-significant within the remote/outer regional sample.

## Discussion

### Overview

To our knowledge, the present study is the first to provide quantitative, national-level evidence on the patterns of SSB consumption by Aboriginal and Torres Strait Islander children in the first years of life. Our exploratory findings highlight some positive elements of beverage intake by young Aboriginal and Torres Strait Islander children: more than one in four children in the sample were still being breast-fed at age >12–18 months and the prevalence of water consumption was high, at 80–90 % across age groups and levels of remoteness. However, there are clear opportunities to improve child nutrition, with a substantial proportion of children across remote, regional and urban settings consuming SSB from an early age, as well as fruit drink, flavoured milk and other forms of milk in the first year of life.

We identified a diverse range of factors associated with the consumption of SSB by young children in LSIC, reflecting the complexity of factors influencing Aboriginal and Torres Strait Islander child nutrition^(^^36^^)^. In general, we observed lower SSB consumption prevalence among children whose families experienced socio-economic advantage, good life circumstances, and good health and well-being, broadly consistent with Australian and international evidence^(^^1^^,^^19^^,^^42^^)^. We observed differences between urban/inner regional and remote/outer regional settings in both the prevalence of SSB consumption and associated factors, demonstrating the importance of environmental and structural factors to children’s nutrition.

### Beverage intake

SSB consumption prevalence was relatively high even within the youngest age group (29·3 %, unadjusted, of children aged 0–12 months) and increased steeply with age (to 56·2 % of children aged 12–18 months and 65·7 % of children aged 18–36 months). Across age groups, of the SSB, cordial consumption was most common (46·8 %), followed by soft drink (19·2 %) and sweetened tea/coffee (12·7 %). Patterns of consumption are generally consistent with those from localised quantitative and qualitative studies^(^^23^^–^^25^^)^ and with national NATSINPAS data, noting differences in outcome definition and age group: 26·0 (95 % CI 13·5, 18·5) % of children aged 2–3 years had consumed cordial the day prior to interview, 17·9 (95 % CI 5·4, 26·5) % soft drinks and 2·7 (95 % CI 0·7, 4·7) % tea^(^^22^^)^. Consumption of fruit drink and flavoured milk was also common among children in the present sample, as has been observed in previous studies^(^^19^^,^^21^^,^^22^^,^^24^^,^^25^^)^. Children’s consumption of fruit drink^(^^24^^,^^25^^,^^43^^)^ and flavoured milk may be encouraged by caregivers who perceive this to be a healthy choice, despite the potentially high sugar content.

One-third (33·6 %, unadjusted) of children up to 12 months of age were still being breast-fed, decreasing to 26·6 % among children aged >12–18 months and 18·1 % among those aged >18–36 months. The prevalence of breast-feeding was substantially higher in remote *v*. urban settings (47·6 *v*. 14·2 %, unadjusted), consistent with previous research^(^^7^^,^^25^^,^^36^^,^^37^^)^. Aligned with this, formula consumption was more common among children living in urban *v*. remote settings (41·4 *v*. 23·9 %, unadjusted)^(^^37^^,^^44^^)^. This may relate to increased continuation of, or reduced disruption to, pre-colonisation feeding practices in more remote settings^(^^34^^,^^37^^)^.

Almost one-third of children up to 12 months of age had consumed cow’s milk (30·0 %, unadjusted). Previous research has identified that there may be a lack of awareness that cow’s milk is not recommended at this age^(^^6^^)^. Other forms of milk, including powdered, long-life, tinned and coconut, were commonly consumed in remote areas, with one in four children (24·2 %, unadjusted) consuming these drinks, compared with 5·8 and 2·3 % in regional and urban areas, respectively. Previous research has identified that some Aboriginal and Torres Strait Islander mothers perceive powdered milk to be beneficial for the baby, particularly mothers who consumed powdered milk when they themselves were growing up^(^^6^^)^ (common on missions and stations^(^^33^^,^^45^^)^). Powdered milk has also been described as an alternative for families who did not have enough money to buy formula^(^^6^^)^.

### Factors associated with sugar-sweetened beverage consumption

#### Remoteness

The prevalence of SSB consumption was significantly lower among children living in major cities (36·3 %; PR = 0·63, 95 % CI 0·49, 0·80) and regional areas (49·7 %, unadjusted; PR = 0·85, 95 % CI 0·68, 1·07) compared with remote areas (62·8 %). Higher consumption in regional and remote settings may reflect affordability- and accessibility-related barriers to recommended food choices in these communities^(^^19^^,^^46^^,^^47^^)^. For example, the price of healthy food in remote communities is significantly higher compared with major cities or regional centres, and there are limited food outlets; this, compounded by lower average socio-economic position in remote settings, may limit families’ ability to make healthy food choices^(^^46^^)^.

In addition, many people living in regional and remote communities in Australia do not have ready access to safe drinking-water^(^^48^^,^^49^^)^. In the focus group, RAOs raised concerns about water quality in many regional and remote settings, citing problems such as yellow bore water, high levels of lead in water and ‘poisoned’ water (such as contamination with per- and polyfluoroalkyl substances in Katherine, Northern Territory^(^^50^^)^). Previous research suggests that tap water is perceived as ‘unhealthy’ in some settings^(^^51^^)^, including due to a history of water quality problems^(^^49^^)^. RAOs explained that, when concerned about water taste or safety, many people avoid drinking tap water and buy bottled water or other beverages; when bottled water is the same price as SSB, people may opt for SSB. This fits with previous research suggesting that the lack of palatable water can lead to high consumption of SSB and other ready-to-drink beverages in Australia^(^^48^^)^ and internationally^(^^52^^)^. For example, research in an Australian^(^^53^^)^ and a Canadian^(^^52^^)^ remote community with poor water quality identified that it was common to mix water with cordial or tea to make it drinkable, and that soft drinks were more commonly consumed than tap water. Redressing water conflicts in Australia could have multiple benefits for Aboriginal and Torres Strait Islander peoples’ well-being, including decreased SSB consumption^(^^48^^,^^54^^,^^55^^)^.

In many regional and remote communities there are frequent disruptions to electricity supply, including families being unable to pay for electricity for periods of time. Without regular electricity supply, it is not possible to cool warm tap water. RAOs highlighted that the warm climate of many remote areas also contributed to high SSB intake, with SSB perceived to quench thirst better than (warm) water. This is supported by previous research demonstrating the role of temperature in beverage selection among Aboriginal and Torres Strait Islander people^(^^53^^)^. These structural and environmental factors linked to the remote/outer regional setting may contribute to the higher prevalence of SSB consumption in regional and remote areas, and may explain why we identified relatively fewer factors associated with SSB intake in the remote/outer regional sample compared with the urban/inner regional sample: individual or family characteristics may be overpowered by community-level factors^(^^47^^)^.

Previous findings from LSIC identified higher consumption of SSB (soft drink, cordial and sports drink combined) among children aged 3–9-years living in more urban compared with more remote settings^(^^56^^)^. This may reflect age variation in the association between remoteness and SSB intake. While SSB consumption prevalence was higher in the remote *v*. urban sample within the younger age groups and overall, the increase with age was more pronounced in the urban/inner regional sample, from 19·6 to 66·1 % (PR = 3·33, 95 % CI 2·40, 4·62; absolute difference = 46·5 %), compared with 41·6 to 65·2 % in the remote/outer regional sample (PR = 1·56, 95 % CI 1·18, 2·06; absolute difference = 23·6 %). Thus, childhood SSB consumption is a concern across settings (online supplementary material, Supplemental Table S1).

#### Other demographic factors

We found that the prevalence of SSB consumption was lower among children with older compared with younger mothers. This may be explained by higher levels of employment or financial security among older compared with younger mothers, or other unmeasured factors such as education. SSB consumption prevalence was significantly lower in households with fewer children. This is consistent with previous research showing an association between smaller household size and recommended child feeding behaviours such as frequency of meals^(^^23^^)^, and may reflect fewer competing pressures on caregivers.

#### Social support

We identified protective associations between multiple forms of caregiver social support (instrumental, social network, emotional) and children’s SSB consumption. This is consistent with local^(^^6^^)^ and international^(^^57^^–^^59^^)^ literature on the importance of social support for optimum infant nutrition. Previous research has identified that many mothers consider family and Elders to be key sources of advice, often preferred over health professionals^(^^60^^)^. This knowledge and informational support can serve as a health resource^(^^32^^,^^60^^)^; however, we found that, within urban settings, caregiver receipt of pregnancy/birthing advice from kin was associated with a significant increase in child SSB consumption prevalence. This might indicate that within urban settings, the advice being shared was not aligned with dietary guidelines. Given the trust placed in advice from kin, building local knowledge and support within the community (rather than only within health providers) may contribute to increased knowledge transfer and improved nutritional outcomes^(^^32^^,^^60^^)^.

#### Socio-economic position and stressors

Socio-economic advantage (area-level advantage, caregiver employment, family financial security) was associated with a lower prevalence of SSB consumption. This aligns with previous findings of socio-economic gradients in SSB intake among older Aboriginal and Torres Strait Islander children (aged 3–9 years)^(^^56^^)^, and early and persistent socio-economic inequalities in sugar-sweetened food and drink intake among young Australian children^(^^16^^,^^17^^,^^61^^)^. SSB intake by those experiencing financial strain may relate to reduced affordability, availability and/or accessibility of recommended drinks and foods^(^^23^^,^^26^^,^^46^^)^. In the context of financial insecurity, the relatively cheaper cost of SSB may encourage their consumption compared with milk, formula, fruit juices, bottled water or milk. We did not observe an association between SSB intake and socio-economic factors within the remote sample; this supports the hypothesis that within remote/outer regional settings, structural and environmental factors related to the remote/outer regional setting itself may have a greater influence on SSB consumption than individual or family-level factors^(^^47^^,^^62^^)^.

We found that children were less likely to consume SSB if their family was not exposed to other forms of stressors, including housing insecurity, discrimination, humbugging or negative major life events. This is consistent with previous research indicating that complex life circumstances or exposure to stressors may make it more difficult for families to provide optimum nutrition for their children^(^^5^^,^^31^^,^^63^^)^. In the focus group, the RAOs echoed that, for many families, there were ‘more important’ things to worry about, so what their children were drinking was not their highest concern. The observed association between discrimination and children’s SSB consumption is consistent with previous LSIC findings of an association between caregivers’ racism experiences and reduced access to nutrition for their children^(^^47^^)^ and qualitative findings identifying a link between current health behaviours and current and historical experiences of discrimination and exclusion^(^^31^^)^.

#### Health provider interactions

We found that SSB consumption prevalence was lower among children whose families engaged in regular prenatal health check-ups and who had adequate access to health services. However, we did not find that postnatal home visits were associated with lower SSB consumption prevalence; compared with families receiving no postnatal visits, families receiving visits from a non-AHW health provider had a similar prevalence of child SSB consumption and families receiving visits from an AHW had a significantly higher prevalence of child SSB consumption. This finding was consistent across the combined, urban/inner regional and remote/outer regional samples. AHW and Aboriginal Community Controlled Health Services are generally understood to provide more relevant and trusted advice and care than non-Aboriginal service providers^(^^31^^,^^32^^,^^60^^,^^63^^,^^64^^)^. As such, we hypothesised that children in families receiving postnatal home visits from AHW would be least likely to consume SSB. There are a number of potential explanations for this contrary finding. In some settings, AHW home visits are targeted at families considered ‘high risk’ according to factors that were independently associated with increased SSB intake in this sample (socio-economic position, stressors or identified barriers to accessing health services). For example, Aboriginal and Torres Strait Islander families in Sydney are recruited into the Maternal Early Childhood Sustained Home-visiting programme if they are classified as high risk according to factors such as young maternal age, experience of major stressors, psychosocial distress, or a current or past mental health problem^(^^65^^)^; mothers participating to the Australian Nurse–Family Partnership Program (ANFPP) in Alice Springs were more likely to be younger and have insecure housing than mothers who were not referred or who declined to participate^(^^66^^)^.

This finding might indicate the benefits of further building capacity in child nutrition among AHW (as well as other health providers). Previous research has identified a need to build nutrition expertise among AHW^(^^5^^,^^29^^)^, including providing AHW clear and contextually relevant information to disseminate to families^(^^29^^,^^67^^)^ and building AHW confidence in delivering correct nutritional messages^(^^5^^)^. Training gaps have also been identified for other health providers working in Aboriginal and Torres Strait Islander infant and child health^(^^5^^,^^6^^,^^63^^)^.

Previous research has identified that Aboriginal and Torres Strait Islander child nutrition is not always viewed as a key issue by health providers^(^^24^^,^^29^^)^, potentially because health-care interactions are focused on other, acute issues perceived to be more pressing^(^^63^^)^. For example, an evaluation of the ANFPP in Alice Springs found that the complexity of families’ lives often reduced both health workers’ ability to provide programme content and mothers’ ability to put messages into practice^(^^67^^)^. The ANFPP evaluation highlighted that it is not feasible to expect an infant home-visiting programme to ‘solve underlying social issues’ within their current time and capacity, and identified the potential value of an additional role dedicated to addressing social determinants of health^(^^67^^)^. Further research could investigate the role and capacity of health services, as well as broader social support services, in improving child nutrition.

#### Maternal and caregiver health behaviours

We hypothesised that SSB intake and breast-feeding would be linked, with children breast-fed for longer durations less likely to consume SSB, as has been observed in international research^(^^42^^)^. However, we did not observe an association between duration of breast-feeding and infant SSB consumption in the present study. This suggests that both mothers who are breast-feeding and mothers who are not breast-feeding require support and education to maintain healthy infant feeding behaviours; efforts are required both to increase breast-feeding duration (particularly in urban areas) and decrease SSB consumption in order to benefit children’s health.

In the current study, children were significantly less likely to consume SSB if they were not exposed to smoke *in utero* and if their caregiver was not a current smoker. We are unaware of any other findings specific to maternal smoking and infant SSB consumption, but we hypothesise that this may reflect non-smoking mothers being ‘health conscious’^(^^68^^,^^69^^)^ or the association between non-smoking and social advantage^(^^70^^)^. Caregivers who smoke might benefit from additional support and advice regarding child nutrition^(^^68^^,^^71^^,^^72^^)^.

#### Well-being

Children were significantly less likely to consume SSB if their caregiver currently had good (compared with poor) social and emotional well-being and physical health. This is consistent with previous qualitative research describing the importance of mental health to parenting^(^^31^^)^ and suggests that approaches to improve child nutrition should be integrated, incorporating supports for caregiver and family well-being^(^^6^^,^^29^^,^^31^^,^^64^^)^. Given the importance of culture to well-being^(^^73^^)^, and given evidence of the healthy infant feeding patterns pre-colonisation, we hypothesised that SSB consumption would be less common among children whose caregivers had a stronger connection to culture. However, we did not observe an association in the present study. It is possible that the variable employed is not a robust indicator of cultural connection or that cultural factors are not directly linked to SSB consumption.

### Limitations

A number of potential limitations should be considered when interpreting these findings. We were limited to examining variables collected in LSIC and relied upon information reported by the caregiver, which may be influenced by recall, reporting or social desirability biases. As a result, these data may under-represent SSB prevalence consumption in the cohort. However, many caregivers did report SSB intake by their children, consistent with previous research, suggesting that social desirability bias was not preventing caregivers from reporting this behaviour. Further, LSIC did not use validated questions to collect data about child SSB consumption. There are few existing dietary questionnaires validated for use with Aboriginal and Torres Strait Islander peoples^(^^18^^,^^74^^)^ and we are unaware of any measures validated for use with this age group. The questions used in the present study do not provide a comprehensive assessment of dietary intake; we focused on SSB intake given community concern and known lack of nutritional benefit. While they are often high in sugar, fruit juice and flavoured milk were not classified as SSB in the present study, as the sugar content of these beverages was not recorded in the survey and there is the potential for these beverages to contain nutritional benefit^(^^75^^,^^76^^)^. However, particularly given their high consumption prevalence, this merits further investigation.

The present study did not explore characteristics of the child’s other caregivers, such as employment status, which may have an important role in children’s diets^(^^77^^,^^78^^)^.

Given the cross-sectional design, we could not determine the direction of associations or make causal inferences. It is possible that observed exposure–outcome associations are confounded by other factors. However, the present analysis was exploratory in nature, quantifying the association between child SSB intake and a broad range of factors; it was not intended to be a predictive model, incorporating all potential confounders. Given the large number of factors examined, it is possible that some of the observed significant associations are due to chance alone. We did not adjust for multiple comparisons in the present study due to its exploratory nature; additional studies are required to confirm results^(^^79^^)^.

Data from LSIC are not intended to be representative of all Aboriginal and Torres Strait Islander children; data on prevalence of SSB intake are representative only of the 933 children in the sample. In contrast, findings from internal comparisons, i.e. the PR calculated in the present study, are intended to be generalisable beyond the study population^(^^84^^,^^85^^)^.

## Conclusion

It is well established that nutrition in the early years is important for health in the short and long term. Consistent with previous findings, the present study provides national evidence that SSB consumption is common among Aboriginal and Torres Strait Islander children from the first year of life. There is international evidence that early childhood (age 2–6 years) is a critical period for the development of sustained obesity^(^^82^^)^. Given the established link between SSB and obesity risk, and the high consumption prevalence of SSB in early childhood in this population, there is potential to promote healthy weight trajectories (as well as other positive health outcomes) through reducing early SSB consumption^(^^83^^)^.

The present exploratory study found that socio-economic advantage, social support, limited exposure to stressors and caregiver well-being were linked to lower SSB consumption prevalence among young Aboriginal and Torres Strait Islander children. These findings reiterate the importance of considering the well-being of the whole family when creating policies and programmes to support healthy diets for Aboriginal and Torres Strait Islander infants and children, rather than focusing on the child in isolation. Coordinated programmes that address these interrelated factors and re-build local nutritional knowledge could lead to improved child nutrition^(^^31^^,^^60^^,^^84^^,^^85^^)^.

Health providers’ advice and information needs to be relevant and applicable to the context of families’ lives, for example considering the range of stressors, competing priorities or barriers that caregivers might face^(^^5^^,^^6^^,^^29^^,^^31^^,^^32^^,^^36^^)^. However, improving information and knowledge addresses only one of the many barriers to optimum child nutrition^(^^18^^,^^29^^)^. Health system strategies need to be paired with upstream strategies, such as holistic support programmes for families^(^^31^^,^^60^^,^^67^^,^^85^^)^, and efforts to reduce racism and improve the food environment, including water quality and safety.

While the present study indicates that SSB consumption is common among young Aboriginal and Torres Strait Islander children, this is not an issue specific to this population^(^^18^^)^; there is clear evidence of a high prevalence of SSB intake among infants in the total Australian population^(^^1^^,^^17^^,^^19^^,^^86^^)^ and in other countries including Finland^(^^87^^)^ and Brazil^(^^88^^)^. Efforts to reduce SSB consumption by Aboriginal and Torres Strait Islander children could be broadened to lead to health benefits at the population level.

## Footnotes

* Some Aboriginal and Torres Strait Islander people use the term ‘humbug’ to refer to demands made by relatives for money or other resources.

## Supplementary material

For supplementary material accompanying this paper visit https://doi.org/10.1017/S1368980019001812. click here to view supplementary material
