# Supplementary material for: Sugar-sweetened beverage consumption among Indigenous Australian children aged 0–3 years and association with sociodemographic, life circumstances and health factors
Source: Public Health Nutr. 2019 Aug 28;23(2):295–308. doi: 10.1017/S1368980019001812 (PMC6988377; doi:10.1017/S1368980019001812)
Supplement: Supplementary file 1 [file S1368980019001812sup.zip › S1368980019001812sup001.docx]

**Supplementary material**

**SUPPLEMENTAL FILE S1: DETAILED EXPLANATION OF EXPOSURES**

All measures relate to the time of the baseline survey, unless otherwise indicated.

**Sociodemographic factors**

Sociodemographic factors included the child’s gender, age group, and identification as Aboriginal, Torres Strait Islander or both; area-level advantage; level of remoteness; the age of the child’s mother at their birth; the caregiver’s relationship and employment status; and the number of children in the household.

***Children’s age*** at the time of interview was categorised as: 0-12 months, >12-18 months or >18-36 months.

***Children’s gender*** was categorised as male or female.

***Child Indigenous status*** was categorised as Aboriginal, Torres Strait Islander, or both Aboriginal and Torres Strait Islander.

***Area Level Advantage*** was measured using the Index of Relative Indigenous Socioeconomic Outcomes (IRISEO) ^(1)^. The IRISEO, specifically designed for Indigenous Australians, includes nine measures of socio-economic outcomes across employment, education, income and housing from the 2006 Census to create a single index for 37 Indigenous regions and 531 Indigenous areas. The lower the decile, the greater the level of disadvantage. In order to be consistent with government reporting, this study used the ASCG Remoteness area classification: ‘most advantaged’ (IRISEO 8–10), ‘mid-level advantage’ (IRISEO 4–7) and ‘most disadvantaged’ (IRISEO 1–3).

***Remoteness*** was measured according to ASCG Remoteness areas 2006: [1] Major Cities of Australia, [2] Inner Regional Australia, [3] Outer Regional Australia, [4] Remote Australia, and [5] Very Remote Australia. Remoteness was categorised as major cities, regional (inner and outer regional), or remote (remote and very remote). When used for stratified analysis, a binary variable of urban/inner regional (major cities and inner regional areas) versus remote/outer regional (outer regional, remote, and very remote areas) was used.

***Mother’s age at the child’s birth*** was categorised as: not birth mother (caregiver was not the child’s birth mother), up to 21 years old, >21-30 years old or >30 years old.

***Caregiver is partnered:***  If caregivers indicated they had a partner in the household, they were classified as partnered.

***Caregiver employment*** was categorised as employed or not employed, according to caregivers’ response to the question, “Do you have a job?”.

***Number of children in the household*** was categorised as 1, 2, 3, or 4 or more.

**Life circumstances factors**

Life circumstances factors included potential supports and stressors. We examined measures of social support: instrumental support (strong family), social network (lots of friends), emotional support (someone you can talk to when upset), and informational support (received advice from friends or family about pregnancy and childbirth). Potential stressors included family financial strain and worries about money; housing security (the number of houses the child had lived in since birth) and housing problems (whether the home needed major repairs); caregiver experiences of discrimination in the past three months; and number of negative major life events; and, whether the family had been humbugged in the past year.

***Instrumental support (caregiver has a strong family)*** was measured according to caregiver responses to the question, “How much is this like you … You have a strong family who help each other?”. The response options were always, most times, sometimes, not really, or other. These were categorised as always, most times, or not really or sometimes.

***Social network (caregiver has lots of friends)*** was measured according to caregiver responses to the question, “How much is this like you … You got lots of friends?”. The response options were always, most times, sometimes, not really, or other. These were categorised as always, most times, or not really or sometimes.

***Emotional support (caregiver has someone to talk to when upset)*** was measured according to caregiver responses to the question, “How much is this like you … When you’re sad or upset you have a person that you can talk to?”. The response options were always, most times, sometimes, not really, or other. These were categorised as always, most times, or not really or sometimes.

***Informational support (caregiver advice from mothers, Aunties, Elders):*** caregivers were asked, “During (your/her) pregnancy with (Study Child) where did (you/she) get information or advice about pregnancy or giving birth from?”. We recorded if caregivers responded that they had received support from mothers and/or Aunties, or from a Traditional healer or community Elder, or if they had not received support from these sources.

***Family financial strain*** was measured according to caregivers’ response to the question, “Which words best describe your family’s money situation?”, with possible response options: we run out of money before payday; we are spending more money than we get; we have just enough money to get us through to the next payday; there’s some money left over each week but we just spend it; we can save a bit every now and then; we can save a lot; or don’t know. Responses were categorized as: we run out of money (we run out of money before payday or we are spending more money than we get), we have just enough money (we have just enough money to get us through to the next payday or there’s some money left over each week but we just spend it), or we can save money (we can save a bit every now and then or we can save a lot).

***Family worries about money:*** Caregivers were asked, “In the last 12 months has your family had serious worries about money?”, to which they responded yes or no.

***Home needs major repairs:*** Caregivers were asked “Does your home have any major things that need fixing? Such as plumbing, fencing, electrical or gas, flooring or kitchen/appliances?”, to which they answered yes or no.

***Number of homes since SC's birth*:** Caregivers were asked, “How many homes has [Study Child] lived in since she/he was born?”. Responses were categorised as 1, 2, or 3 or more houses.

***Caregiver experienced discrimination*:** Caregivers were asked “Have you been treated unfairly or discriminated against (treated badly) because you are Aboriginal/Torres Strait Islander?”. Response options were not really, a little bit, a fair bit, or lots of times. These responses were grouped into three categories: not really, or a little bit to lots of times. Caregivers who did not identify as Aboriginal or Torres Strait Islander were not asked this question, and responses are coded as missing. As a result, the missing category is large for this variable.

***Humbugged in the last year:*** Caregivers were asked, “In the last year have you or your family been humbugged?”, to which they responded yes or no. Some Aboriginal and Torres Strait Islander people use the term ‘humbug’ to refer to demands made by relatives for money or other resources ^(2)^.

***Number of negative life events:*** Caregivers were asked if they had experienced a set of major life events in the past year. We examined responses to the following ten questions about negative life events:

1. In the last year, have you or a close family member been badly hurt or sick?
2. In the last year, has a close family member or friend passed away?
3. In the last year did you, or another of (Study Child)’s carers lose their job?
4. In the last year have you or a close family member had an alcohol or drug problem?
5. In the last year have you or a close family member been mugged, robbed or assaulted?
6. In the last year have you or a close family member been arrested, been in jail/prison, or had problems with the police?
7. In the last year has (Study Child) or any other child of yours been involved in or upset by family arguments?
8. In the last year has (Study Child) or any other child of yours been badly scared by other people’s behaviour?
9. In the last year have any of (Study Child)’s parents or carers left because of a family split-up?
10. In the last year has (Study Child) or any other child of yours had to be cared for by someone else for a while (at least a week)?

The number of yes responses to the above questions were summed, and categorised as 0, 1, 2, or 3-10 negative life events.

**Health and wellbeing factors**

Health and wellbeing factors included measures of health provider interactions, health behaviours, and wellbeing during and after the child’s birth. We examined whether the child’s mother had regular pre-natal check-ups or smoked during the pregnancy. We examined the duration of breastfeeding with the child; if the mother experienced postnatal depression after the child’s birth; and the caregiver’s social and emotional wellbeing, and child and caregiver general health, at the time of the survey. We examined the child’s access to healthcare and if the family received postnatal home visits by a health professional. We also examined the caregiver’s current smoking status and knowledge about Indigenous family, culture, and history (cultural knowledge).

***Pre-natal check-ups:*** Caregivers were asked, “Did [you/study child’s mother] have any check-ups during [your/her] pregnancy with [study child]?” and, “Did [you/study child’s mother] have regular check-ups during the pregnancy, that is, at least 1 check-up every 2 months?]. Responses to these two questions were combined to form three categories: no check-ups, some check-ups but not regular, and regular check-ups.

***Caregiver smoked during pregnancy*** was measured according to caregivers’ response to the question, “After finding out [you/Study Child’s mother] were pregnant with [Study Child], did [you/she] smoke any cigarettes during the pregnancy (including chewing tobacco)?”.

***Duration of breastfeeding:*** Caregivers were asked, “Was [Study Child] ever breastfed”, “Is [Study Child] still being breastfed?”, and “How old was [Study Child] when he/she completely stopped being breastfed?”. Duration of breastfeeding was calculated according to responses to these questions, and categorised as: never breastfed, breastfed for less than six months, and breastfed for 6 months or more. Children who were less than 6 months old at the time of survey (n<10) were categorised as either never breastfed (if no breastfeeding was reported) or breastfed for less than six months (if any breastfeeding was reported).

***Post-natal depression:*** Caregivers were asked, “Did [you/the Study Child’s mother] suffer from baby blues or post-natal depression for at least a month? For example, did [you/she] feel really down for more than a month?”, with possible response options: yes, probably, no or don’t know. If caregivers responded yes or probably, they were then asked, “Did [you/Study Child’s mother] get treatment for this?”, with possible response options yes, no or don’t know. Responses to these questions were combined to produce three categories: no post-natal depression; yes post-natal depression (treated); and, yes post-natal depression (not treated).

**Caregiver social and emotional wellbeing**: LSIC includes a set of seven questions that can capture subjective wellbeing ^(3)^. Unfortunately these seven questions do not capture positive aspects of emotional wellbeing, which is an important part of wellbeing. This social and emotional wellbeing index includes questions related to depression (including anger and impulsivity) and anxiety, and can provide an indication of the level of negative emotional wellbeing ^(3)^. Carers are asked if, in the last three months, they:

1. Have you stopped liking things that used to be fun?
2. Have you felt like everything is hard work (even little jobs are too much)?
3. Have you felt so worried that your stomach (tummy) has got upset?
4. Have you ever felt so worried it was hard to breathe?
5. Do you get angry or wild real quick?
6. Have you felt so sad that nothing could cheer you up? Not even your friends made you feel better.
7. Do you do silly things without thinking that you feel ashamed about the next day?

Response options were: never (or not much), little bit (or sometimes), fair bit, lots (or lots of times), or don’t know. The first two responses (never of little bit) were coded as 0 and the latter two (fair bit or lots) as 1 ^(4)^. Scores for the seven questions were summed (range: 0-7), with higher scores reflecting better social and emotional wellbeing. Carers were categorised as having a good wellbeing (lower distress, score 0-1) or poor wellbeing (higher distress, score 2-7).

***Child general health:*** Caregiver’s were asked, “In general, would you say child’s health is excellent, very good, good, fair or poor?”. Responses were grouped into two categories: good, fair, or poor, or excellent or very good.

***Caregiver general health:*** Caregivers were asked, “In general, would you say your health is excellent, very good, good, fair or poor?”. Responses were grouped into 2 categories: good, fair, or poor, or excellent or very good.

***Access to health services*** was measured according to caregivers’ response to the question, “Has there been a time when study child needed to see a doctor, but didn’t go for some reason?”. Responses of “yes” were considered an indicator of an unmet need for health service; responses of “no” were considered an indicator of adequate access to health services.

***Home visits after birth:*** Caregivers were asked, “Since the child’s birth, have you had a home visit from a: nurse, mid-wife, social worker, Aboriginal Health Worker (AHW) or cultural consultant, general practitioner or any other visits?”. Responses were grouped as: no home visits; home visits, but none from an AHW; or home visits, including any from an AHW.

***Caregiver currently smokes:*** Caregivers were asked, “Do you currently smoke or chew tobacco?”, to which they responded yes or no.

***Caregiver knows about Indigenous family, culture and history*** was measured according to caregiver response to the question, “How much is this like you … You know a lot about your Aboriginal (Torres Strait Islander) family history and culture?” The prompt was re-worded to “You understand a lot about Aboriginal or Torres Strait Islander history or culture?” for non-Indigenous caregivers. Response options were lots, fair bit, little bit, not much, other, don’t know, or refusal. Responses were categorised as: not much or a little bit, a fair bit, or lots.

**SUPPLEMENTAL FILE S2: RESULTS OF ANALYSIS STRATIFIED BY REMOTENESS**

*Socio-demographic factors.* Within the urban/inner regional sample, the prevalence of SSB consumption among urban/inner regional children increased steeply with age (p-trend<0.05, up to PR=3.33; 95%CI:2.40,4.62 for children aged >18-36 versus 0-12 months) and decreased with increasing advantage (p-trend<0.05, down to PR=0.56; 0.37,0.85) (Table SI1). SSB consumption prevalence was lower among children with older versus younger mothers (PR=0.74; 0.58,0.94 and PR=0.60; 0.48,0.75 for mothers aged 21-30 and >30 years, respectively, at the child’s birth), and among children whose caregiver was employed (PR=0.67; 0.53,0.85). There was not a significant association between SSB consumption prevalence and child gender, child Indigenous status, caregiver relationship status, or number of children in the household within the urban/inner regional sample.

Within the remote/outer regional sample, SSB consumption prevalence increased with age (p-trend<0.05, up to PR=1.56; 1.18,2.06), though the gradient was less steep (p-interaction<0.05 for both categorical and continuous variable), and was significantly lower in households with one child (PR=0.79; 0.62,0.99) compared to four or more children. SSB consumption prevalence within the remote/outer regional sample was not significantly associated with any other sociodemographic factors (Table SI1).

*Life circumstances factors*. Within the urban/inner regional sample, SSB consumption prevalence was significantly higher among children whose caregiver received versus did not receive advice from friends or family (PR=1.29; 1.09,1.52) (Table SI1). SSB consumption prevalence was significantly associated with the caregiver’s social network (overall p-value=0.005), but was not significantly associated with social network or emotional support. SSB consumption prevalence was significantly lower among children whose family did not experience financial strain (PR=0.70; 0.58,0.85 and PR=0.52; 0.41,0.66 for families with just enough money and families who could save, respectively, versus families who run out of money) or have worries about money (PR=0.72; 0.58,0.88), who had a stable home environment (PR=0.73; 0.56,0.96 for children living in the same home, versus 3 or more homes, since birth), and families who were not humbugged in the last year (PR=0.76; 0.58,0.98). SSB consumption prevalence did not vary significantly by number of children in the household, but decreased with decreasing number of negative major life events (p-trend<0.05, down to PR=0.50; 0.35,0.71 for families experiencing 0 compared to 3-10 events). We did not observe a significant association in the urban/inner regional sample between SSB consumption prevalence and the caregiver’s relationship status or experiences of discrimination, or the home’s need for major repairs.

Within the remote/outer regional sample, there was not a significant association between SSB consumption prevalence and any of the indicators of social support; this resulted in a significant interaction by remoteness for informational support (advice from family/friends), which was significant within the urban/inner regional sample. SSB consumption prevalence was significantly lower among children whose caregivers had limited experience of discrimination (PR=0.87; 0.76,0.99) and whose families had not been humbugged in the past year (PR=0.83; 0.72,0.96), but was not associated with any other measure of life circumstances.

*Health and wellbeing factors.* SSB consumption prevalence was significantly lower among children unexposed versus exposed to smoke in utero (PR=0.65; 0.53,0.80), whose caregivers had good versus poor social and emotional wellbeing (PR=0.63; 0.50,0.79), who had adequate health service access versus an unmet need for health service in the past year (PR=0.61; 0.47,0.81), and whose caregiver was not a current smoker (PR=0.61; 0.47,0.81) (Table SI1). SSB consumption prevalence was significantly higher among children whose families received postnatal home visits from an AHW (PR=1.37; 1.01,1.85), but not for children who received home visits from another service provider (PR=0.92; 0.71,1.20), compared to no home visits. We did not observe a significant association between SSB consumption prevalence and pre-natal check-ups, breastfeeding duration, post-natal depression, child or caregiver physical health, or the caregiver’s cultural knowledge.

Within the remote/outer regional sample, SSB consumption prevalence was significantly lower among children whose caregiver had good versus poor social and emotional wellbeing (PR=0.79; 0.67,0.94). Compared to children who received no postnatal home visits, SSB consumption prevalence was significantly higher among children who visited by AHWs (PR=1.40; 1.18,1.66) but not by other service providers (PR=1.07; 0.89,1.28).

**Supplemental Table S1: Association between children’s SSB consumption and sociodemographic, life circumstances, and health and wellbeing factors, stratified by remoteness**

|  | **URBAN/INNER REGIONAL** | | | | |  | **REMOTE/OUTER REGIONAL** | | | | |
| --- | --- | --- | --- | --- | --- | --- | --- | --- | --- | --- | --- |
|  | **% any SSB** | **n/N** | **PR** | **95% CI** | **P-value** |  | **% any SSB** | **n/N** | **PR** | **95% CI** | **P-value** |
|  |  |  |  |  |  |  |  |  |  |  |  |
| **Total** | 39.9 | 192/481 | -- | -- | -- |  | 62.2 | 281/452 | -- | -- | -- |
| **Sociodemographic factors** |  |  |  |  |  |  |  |  |  |  |  |
| **Child gender** |  |  |  |  | 0.337 |  |  |  |  |  | 0.143 |
| Male | 40.2 | 92/229 | 1 | (Ref) |  |  | 59.0 | 144/244 | 1 | (Ref) |  |
| Female | 39.7 | 100/252 | 0.93 | (0.79,1.08) |  |  | 65.9 | 137/208 | 1.1 | (0.97,1.25) |  |
| **Child age group ***^†‡^ |  |  |  |  | <0.001 |  |  |  |  |  | <0.001 |
| 0-12 months | 19.6 | 31/158 | 1 | (Ref) |  |  | 41.6 | 52/125 | 1 | (Ref) |  |
| >12-18 months | 39.7 | 79/199 | 2.03 | (1.40,2.93) |  |  | 73.5 | 139/189 | 1.75 | (1.36,2.26) |  |
| >18-36 months | 66.1 | 82/124 | 3.33 | (2.40,4.62) |  |  | 65.2 | 90/138 | 1.56 | (1.18,2.06) |  |
| **Child Indigenous status** |  |  |  |  | 0.066 |  |  |  |  |  | 0.107 |
| Aboriginal | 40.4 | 182/451 | 1 | (Ref) |  |  | 65.8 | 237/360 | 1 | (Ref) |  |
| Torres Strait Islander | 21.1 | 4/19 | 0.60 | (0.28,1.27) |  |  | 41.5 | 17/41 | 0.66 | (0.41,1.06) |  |
| Aboriginal & Torres Strait Islander | 54.5 | 6/11 | 1.59 | (0.97,2.59) |  |  | 52.9 | 27/51 | 0.82 | (0.66,1.02) |  |
| **Area-level advantage ^†^** |  |  |  |  | 0.022 |  |  |  |  |  | 0.645 |
| Most advantaged | 31.2 | 49/157 | 0.56 | (0.37,0.85) |  |  | 66.7 | 26/39 | 1.04 | (0.76,1.42) |  |
| Mid-level advantage | 43.6 | 137/314 | 0.73 | (0.49,1.08) |  |  | 59.9 | 161/269 | 0.91 | (0.70,1.20) |  |
| Most disadvantaged | 60.0 | 6/10 | 1 | (Ref) |  |  | 65.3 | 94/144 | 1 | (Ref) |  |
| **Remoteness** |  |  |  |  | 0.383 |  |  |  |  |  | 0.982 |
| Major cities | 36.3 | 93/256 | 1 | (Ref) |  |  | -- | -- | -- | -- |  |
| Regional | 44.0 | 99/225 | 1.14 | (0.85,1.53) |  |  | 60.3 | 73/121 | 1.00 | (0.77,1.31) |  |
| Remote | -- | -- | -- | -- |  |  | 62.8 | 208/331 | 1 | (Ref) |  |
| **Mother’s age at birth** |  |  |  |  | <0.001 |  |  |  |  |  | 0.186 |
| Not birth mother | 44.8 | 13/29 | 0.80 | (0.56,1.13) |  |  | 57.7 | 15/26 | 0.84 | (0.60,1.17) |  |
| Up to 21 years | 56.0 | 51/91 | 1 | (Ref) |  |  | 67.8 | 78/115 | 1 | (Ref) |  |
| 21-30 years | 38.1 | 83/218 | 0.74 | (0.58,0.94) |  |  | 63.1 | 130/206 | 0.92 | (0.81,1.05) |  |
| >30 years | 31.5 | 45/143 | 0.60 | (0.48,0.75) |  |  | 55.2 | 58/105 | 0.81 | (0.66,1.00) |  |
| **Caregiver is partnered *** |  |  |  |  | 0.075 |  |  |  |  |  | 0.232 |
| No | 43.6 | 95/218 | 1 | (Ref) |  |  | 59.2 | 113/191 | 1 | (Ref) |  |
| Yes | 36.9 | 97/263 | 0.85 | (0.71,1.02) |  |  | 64.4 | 168/261 | 1.09 | (0.94,1.27) |  |
| **Caregiver is employed *** |  |  |  |  | 0.001 |  |  |  |  |  | 0.655 |
| No | 44.0 | 148/336 | 1 | (Ref) |  |  | 63.2 | 216/342 | 1 | (Ref) |  |
| Yes | 31.0 | 44/142 | 0.67 | (0.53,0.85) |  |  | 59.4 | 63/106 | 0.96 | (0.80,1.15) |  |
| Missing | 0.0 | 0/3 | -- | -- |  |  | 50.0 | 2/4 | -- | -- |  |
| **Number of children in the household** |  |  |  |  | 0.381 |  |  |  |  |  | 0.062 |
| 1 | 36.8 | 56/152 | 0.79 | (0.59,1.06) |  |  | 52.0 | 53/102 | 0.79 | (0.62,0.99) |  |
| 2 | 39.2 | 51/130 | 0.79 | (0.58,1.08) |  |  | 58.7 | 61/104 | 0.86 | 0.71,1.04) |  |
| 3 | 38.8 | 40/103 | 0.83 | (0.59,1.15) |  |  | 69.5 | 66/95 | 1.02 | (0.87,1.19) |  |
| 4 or more | 46.9 | 45/96 | 1 | (Ref) |  |  | 66.9 | 101/151 | 1 | (Ref) |  |
|  |  |  |  |  |  |  |  |  |  |  |  |
| **Life circumstances** |  |  |  |  |  |  |  |  |  |  |  |
| **Caregiver has a strong family** |  |  |  |  | 0.395 |  |  |  |  |  | 0.388 |
| Not really or sometimes | 46.2 | 30/65 | 1 | (Ref) |  |  | 69.6 | 48/69 | 1 | (Ref) |  |
| Most times | 34.5 | 29/84 | 0.79 | (0.57,1.11) |  |  | 60.0 | 27/45 | 0.85 | (0.66,1.09) |  |
| Always | 40.0 | 132/330 | 0.90 | (0.67,1.21) |  |  | 61.0 | 206/338 | 0.89 | (0.74,1.07) |  |
| Missing | 50.0 | 1/2 | -- | -- |  |  | -- | -- | -- | -- |  |
| **Caregiver has lots of friends** |  |  |  |  | 0.005 |  |  |  |  |  | 0.170 |
| None or not many | 43.2 | 41/95 | 1 | (Ref) |  |  | 66.2 | 51/77 | 1 | (Ref) |  |
| Fair few | 44.6 | 87/195 | 1.02 | (0.76,1.37) |  |  | 66.9 | 83/124 | 1.05 | (0.89,1.23) |  |
| Lots | 33.5 | 64/191 | 0.76 | (0.56,1.03) |  |  | 58.6 | 147/251 | 0.88 | (0.74,1.05) |  |
| **Caregiver has someone to talk to when upset** |  |  |  |  | 0.364 |  |  |  |  |  | 0.189 |
| Never or little bit | 50.7 | 35/69 | 1 | (Ref) |  |  | 67.6 | 48/71 | 1 | (Ref) |  |
| Fair bit | 36.8 | 25/68 | 0.78 | (0.52,1.17) |  |  | 64.8 | 35/54 | 0.97 | (0.81,1.17) |  |
| Always | 38.3 | 131/342 | 0.80 | (0.58,1.10) |  |  | 60.4 | 197/326 | 0.89 | (0.78,1.02) |  |
| Missing | 50.0 | 1/2 | -- | -- |  |  | 100.0 | 1/1 | -- | -- |  |
| **Caregiver advice from mothers, Aunties, Elders *** |  |  |  |  | 0.003 |  |  |  |  |  | 0.122 |
| No | 35.4 | 114/322 | 1 | (Ref) |  |  | 66.2 | 184/278 | 1 | (Ref) |  |
| Yes | 49.3 | 73/148 | 1.29 | (1.09,1.52) |  |  | 57.7 | 75/130 | 0.87 | (0.74,1.04) |  |
| Missing | 45.5 | 5/11 | -- | -- |  |  | 50.0 | 22/44 | -- | -- |  |
| **Family financial strain *** |  |  |  |  | <0.001 |  |  |  |  |  | 0.508 |
| We run out of money | 61.4 | 51/83 | 1 | (Ref) |  |  | 67.6 | 50/74 | 1 | (Ref) |  |
| We have just enough money | 40.2 | 92/229 | 0.70 | (0.58,0.85) |  |  | 65.6 | 124/189 | 0.97 | (0.81,1.18) |  |
| We can save | 28.5 | 45/158 | 0.52 | (0.41,0.66) |  |  | 60.7 | 99/163 | 0.89 | (0.73,1.09) |  |
| Missing | 36.4 | 4/11 | -- | -- |  |  | 30.8 | 8/26 | -- | -- |  |
| **Family worries about money *** |  |  |  |  | 0.002 |  |  |  |  |  | 0.527 |
| No | 34.5 | 107/310 | 0.72 | (0.58,0.88) |  |  | 61.2 | 208/340 | 0.95 | (0.81,1.12) |  |
| Yes | 50.0 | 85/170 | 1 | (Ref) |  |  | 65.3 | 66/101 | 1 | (Ref) |  |
| Missing | 0.0 | 0/1 | -- | -- |  |  | 63.6 | 7/11 | -- | -- |  |
| **Home needs major repairs** |  |  |  |  | 0.148 |  |  |  |  |  | 0.841 |
| No | 37.3 | 122/327 | 0.85 | (0.68,1.06) |  |  | 61.2 | 137/224 | 0.98 | (0.83,1.17) |  |
| Yes | 45.6 | 68/149 | 1 | (Ref) |  |  | 62.5 | 135/216 | 1 | (Ref) |  |
| Missing | 40.0 | 2/5 | -- | -- |  |  | 75.0 | 9/12 | -- | -- |  |
| **Number of homes since SC's birth *** |  |  |  |  | 0.046 |  |  |  |  |  | 0.979 |
| 1 | 34.8 | 112/322 | 0.73 | (0.56,0.96) |  |  | 61.6 | 178/289 | 1.03 | (0.81,1.30) |  |
| 2 | 46.6 | 54/116 | 0.87 | (0.62,1.22) |  |  | 65.1 | 71/109 | 1.02 | (0.81,1.28) |  |
| 3 or more | 61.5 | 24/39 | 1 | (Ref) |  |  | 59.2 | 29/49 | 1 | (Ref) |  |
| Missing | 50.0 | 2/4 | -- | -- |  |  | 60.0 | 3/5 | -- | -- |  |
| **Caregiver experience of discrimination** |  |  |  |  | 0.229 |  |  |  |  |  | 0.031 |
| Not really | 38.8 | 94/242 | 0.86 | (0.68,1.10) |  |  | 62.1 | 187/301 | 0.87 | (0.76,0.99) |  |
| Little bit to lots of times | 46.2 | 55/119 | 1 | (Ref) |  |  | 73.6 | 78/106 | 1 | (Ref) |  |
| Missing | 35.8 | 43/120 | -- | -- |  |  | 35.6 | 16/45 | -- | -- |  |
| **Humbugged in the last year** |  |  |  |  | 0.036 |  |  |  |  |  | 0.014 |
| No | 38.3 | 152/397 | 0.76 | (0.58,0.98) |  |  | 58.2 | 191/328 | 0.83 | (0.72,0.96) |  |
| Yes | 47.6 | 40/84 | 1 | (Ref) |  |  | 72.3 | 86/119 | 1 | (Ref) |  |
| Missing | -- | -- | -- | -- |  |  | 80.0 | 4/5 | -- | -- |  |
| **Number of negative life events ***^†^ |  |  |  |  | <0.001 |  |  |  |  |  | 0.126 |
| 0 | 25.9 | 22/85 | 0.50 | (0.35,0.71) |  |  | 58.7 | 37/63 | 0.85 | (0.68,1.06) |  |
| 1 | 33.6 | 43/128 | 0.62 | (0.50,0.77) |  |  | 58.6 | 58/99 | 0.85 | (0.72,1.00) |  |
| 2 | 40.2 | 39/97 | 0.77 | (0.56,1.06) |  |  | 57.3 | 43/75 | 0.84 | (0.70,1.00) |  |
| 3 to 10 | 52.1 | 85/163 | 1 | (Ref) |  |  | 70.5 | 117/166 | 1 | (Ref) |  |
| Missing | 37.5 | 3/5 | -- | -- |  |  | 53.1 | 26/49 | -- | -- |  |
| **Health and wellbeing** |  |  |  |  |  |  |  |  |  |  |  |
| **Pre-natal check-ups** |  |  |  |  | 0.167 |  |  |  |  |  | 0.490 |
| No check-ups | 62.5 | 5/8 | 1 | (Ref) |  |  | 68.8 | 11/16 | 1 | (Ref) |  |
| Some check-ups, not regular | 56.3 | 9/16 | 0.68 | (0.37,1.27) |  |  | 67.9 | 19/28 | 0.98 | (0.70,1.38) |  |
| Regular check-ups | 38.5 | 171/444 | 0.60 | (0.34,1.03) |  |  | 63.1 | 229/363 | 0.89 | (0.70,1.14) |  |
| Missing | 53.9 | 7/13 | -- | -- |  |  | 48.9 | 22/45 | -- | -- |  |
| **Smoking during pregnancy *** |  |  |  |  | <0.001 |  |  |  |  |  | 0.130 |
| No | 31.4 | 75/239 | 0.65 | (0.53,0.80) |  |  | 59.1 | 101/171 | 0.89 | (0.77,1.03) |  |
| Yes | 48.8 | 104/213 | 1 | (Ref) |  |  | 67.3 | 152/226 | 1 | (Ref) |  |
| Missing | 44.8 | 13/29 | -- | -- |  |  | 50.9 | 28/55 | -- | -- |  |
| **Duration of breastfeeding *** |  |  |  |  | 0.061 |  |  |  |  |  | 0.360 |
| Never breastfed | 44.2 | 53/120 | 1 | (Ref) |  |  | 50.8 | 31/61 | 1 | (Ref) |  |
| Less than 6 months | 43.1 | 59/137 | 1.07 | (0.84,1.35) |  |  | 63.3 | 38/60 | 1.26 | (0.88,1.81) |  |
| 6 months or more | 34.5 | 76/220 | 0.81 | (0.64,1.01) |  |  | 64.0 | 210/328 | 1.26 | (0.92,1.75) |  |
| Missing | 100.0 | 4/4 | -- | -- |  |  | 66.7 | 2/3 | -- | -- |  |
| **Post-natal depression** |  |  |  |  | 0.106 |  |  |  |  |  | 0.995 |
| No | 38.1 | 142/373 | 0.87 | (0.63,1.19) |  |  | 63.7 | 225/353 | 0.99 | (0.72,1.37) |  |
| Yes (treated) | 52.3 | 23/44 | 1.16 | (0.80,1.70) |  |  | 61.9 | 13/21 | 0.98 | (0.60,1.60) |  |
| Yes (untreated) | 40.0 | 18/45 | 1 | (Ref) |  |  | 63.0 | 17/27 | 1 | (Ref) |  |
| Missing | 47.4 | 9/19 | -- | -- |  |  | 51.0 | 26/51 | -- | -- |  |
| **Caregiver social and emotional wellbeing** |  |  |  |  | <0.001 |  |  |  |  |  | 0.007 |
| Good (low distress) | 34.5 | 129/374 | 0.63 | (0.50,0.79) |  |  | 59.7 | 157/263 | 0.79 | (0.67,0.94) |  |
| Poor (high distress) | 56.5 | 52/92 | 1 | (Ref) |  |  | 75.3 | 61/81 | 1 | (Ref) |  |
| Missing | 73.3 | 11/15 | -- | -- |  |  | 58.3 | 63/108 | -- | -- |  |
| **Child general health** |  |  |  |  | 0.701 |  |  |  |  |  | 0.464 |
| Good, fair or poor | 36.5 | 38/104 | 1 | (Ref) |  |  | 67.4 | 60/89 | 1 | (Ref) |  |
| Excellent or very good | 41.0 | 154/376 | 1.06 | (0.80,1.39) |  |  | 61.0 | 221/362 | 0.94 | (0.79,1.11) |  |
| Missing | 0.0 | 0/1 | -- | -- |  |  | 0.0 | 0/1 | -- | -- |  |
| **Caregiver general health** |  |  |  |  | 0.227 |  |  |  |  |  | 0.078 |
| Good, fair or poor | 43.4 | 108/249 | 1 | (Ref) |  |  | 66.4 | 146/220 | 1 | (Ref) |  |
| Excellent or very good | 36.4 | 84/231 | 0.87 | (0.69,1.09) |  |  | 59.3 | 131/221 | 0.86 | (0.73,1.02) |  |
| Missing | 0.0 | 0/1 | -- | -- |  |  | 36.4 | 4/11 | -- | -- |  |
| **Access to health services *** |  |  |  |  | <0.001 |  |  |  |  |  | 0.332 |
| Adequate access | 37.4 | 164/438 | 0.61 | (0.47,0.81) |  |  | 61.2 | 251/410 | 0.89 | (0.70,1.13) |  |
| Unmet need for health service | 64.3 | 27/42 | 1 | (Ref) |  |  | 71.8 | 28/39 | 1 | (Ref) |  |
| Missing | 100.0 | 1/1 | -- | -- |  |  | 66.7 | 2/3 | -- | -- |  |
| **Home visits after birth** |  |  |  |  | <0.001 |  |  |  |  |  | <0.001 |
| No home visits | 39.8 | 45/113 | 1 | (Ref) |  |  | 58.7 | 142/242 | 1 | (Ref) |  |
| Home visits not from AHW | 37.5 | 115/307 | 0.92 | (0.71,1.20) |  |  | 62.1 | 95/153 | 1.07 | (0.89,1.28) |  |
| Home visits from AHW | 58.2 | 32/55 | 1.37 | (1.01,1.85) |  |  | 82.7 | 43/52 | 1.40 | (1.18,1.66) |  |
| Missing | 0.0 | 0/6 | -- | -- |  |  | 20.0 | 1/5 | -- | -- |  |
| **Caregiver currently smokes *** |  |  |  |  | <0.001 |  |  |  |  |  | 0.031 |
| No | 30.4 | 69/227 | 0.64 | (0.52,0.79) |  |  | 54.5 | 90/165 | 0.83 | (0.70,0.98) |  |
| Yes | 48.4 | 123/254 | 1 | (Ref) |  |  | 66.6 | 191/287 | 1 | (Ref) |  |
| **Caregiver knowledge of family, culture, history** |  |  |  |  | 0.624 |  |  |  |  |  | 0.997 |
| Not much or a little bit | 41.5 | 80/193 | 1 | (Ref) |  |  | 63.1 | 70/111 | 1 | (Ref) |  |
| Fair bit | 39.7 | 54/136 | 0.94 | (0.73,1.20) |  |  | 61.9 | 70/113 | 1.00 | (0.81,1.24) |  |
| Lots | 38.7 | 58/150 | 0.88 | (0.68,1.14) |  |  | 61.8 | 141/228 | 1.00 | (0.80,1.23) |  |
| Missing | 0.0 | 0/2 | -- | -- |  |  | -- | -- | -- | -- |  |

*SSB, sugar-sweetened beverages. PR, Prevalence Ratio. All models exclude children missing data on the exposure of interest; all models are adjusted for age group and remoteness (where appropriate). P-value shown is for Wald statistic. The missing category for caregiver discrimination is large as caregivers were only asked this question if they identified as Aboriginal and/or Torres Strait Islander. * Significant interaction by remoteness.* ***^†^*** *Significant trend (p-value for trend <0.05) in urban/inner regional areas and* ***^‡^*** *significant trend in remote/outer regional areas; only tested for ordinal variables. Urban/inner regional includes major cities and inner regional areas; remote/outer regional includes outer regional, remote, and very remote areas.*

**References**

[1] Biddle N. (2009) Ranking Regions-Revisiting an Index of Relative Indigenous Socio-economic Outcomes. Australasian Journal of Regional Studies, The. 15: 329.

[2] Johnston FH, Jacups SP, Vickery AJ et al. (2007) Ecohealth and Aboriginal testimony of the nexus between human health and place. EcoHealth. 4: 489-99.

[3] Biddle N. (2011) An exploratory analysis of the Longitudinal Survey of Indigenous Children. CAEPR Working Paper No. 77/2011. Centre for Aboriginal Economic Policy Research.

[4] Australian Government Department of Families Housing Community Services and Indigenous Affairs. (2013) Appendix B - Study terminology and definitions. Footprints in Time The Longitudinal Study of Indigenous Children: Key Summary Report from Wave 4. Canberra.
